# Correlation analysis of serum SIRT4, CTRP5, and galectin-3 levels with the prognosis of diabetic retinopathy patients

**DOI:** 10.5937/jomb0-61802

**Published:** 2026-06-16

**Authors:** Wenming Cheng, Hua Lu, Song Ting, Wei Liu

**Affiliations:** 1 Yangzhou University School of Medicine, No.136 Jiangyang Middle Road, Yangzhou, China; 2 Taizhou Hospital of Integrated Traditional Chinese and Western Medicine. No. 111, Department of Ophthalmology, Jiangzhou South Road, Hailing District,Taizhou, China; 3 Department of Endocrinology, Taizhou Hospital of Integrated Traditional Chinese and Western Medicine, No. 111, Jiangzhou South Road, Hailing District, Taizhou, China; 4 Endocrinology Department, West China Hospital of Sichuan University, No. 37, Guoxue Lane, Wuhou District, Chengdu, China; 5 Department of Ophthalmology,The Affiliated Suqian First People's Hospital of Nanjing Medical University, No.120 suzhi Road, Suqian, China

**Keywords:** diabetic retinopathy, silent information moderating factor 4, complement C1q tumour necrosis factor-related protein 5, galectin-3, glycolipid metabolism, dijabetička retinopatija, regulator utišavanja informacija 4, protein povezan sa faktorom tumorske nekroze C1q (CTRP5), galektin-3, metabolizam glikolipida

## Abstract

**Background:**

To explore the relationships between serum silencing information regulator 4 (SIRT4), complement C1q tumour necrosis factor-related protein 5 (CTRP5), galectin-3 and glycolipid metabolism and prognosis in patients with diabetic retinopathy (DR).

**Methods:**

The DR group was selected from among 115 hospitalised DR patients admitted between January 2023 and January 2024, including 61 non-proliferative DR patients (Non-proliferative DR group) and 54 proliferative DR patients (Proliferative DR group). Additionally, 50 subjects who underwent health check-ups in the hospital during the same period were selected as the control group. Indicators of SIRT4, CTRP5, galectin-3, blood glucose [Fasting plasma glucose (FPG)], and the levels of blood lipids in the DR group and the control group were measured and compared. Triglycerides (TG), low-density lipoprotein cholesterol (LDL-C), high-density lipoprotein cholesterol (HDL-C), and total cholesterol (TC) were among them. Moreover, the correlations between serum SIRT4, CTRP5, and galectin-3 levels and blood glucose and lipid indicators in DR patients were analysed. The DR patients were monitored and followed up with for six months after treatment. Depending on their level of visual impairment, the patients were split into groups with excellent and poor prognoses. The levels of serum SIRT4, CTRP5 and galectin-3 in the two groups were compared. Multivariate logistic regression was used to analyse the risk factors for poor prognosis in DR patients. A receiver operating characteristic (ROC) curve was used to analyse the predictive value of single or combined detection of SIRT4, CTRP5, and galectin-3 for poor prognosis in patients with DR.

**Results:**

Pearson correlation analysis revealed that the levels of SIRT4, CTRP5, and galectin-3 in DR patients were positively correlated with FPG, TG, TC, and LDL-C (P&lt; 0.05) and negatively correlated with HDL-C (P&lt; 0.05). The poor-prognosis group had higher serum levels of SIRT4, CTRP5, and galectin-3 than the good-prognosis group (P&lt; 0.05). The poor prognosis group's DR course was longer than the excellent prognosis group's (P&lt; 0.05), and the proportion of proliferative DR was greater than that in the good prognosis group (P&lt; 0.05). DR course S6 months, SIRT4 S24 ng/mL, CTRP5 S8 ng/mL, galectin-3&gt; 1,400 ng/mL, and DR stage of the proliferative type were all independent risk factors for poor prognosis in DR patients. The ROC curve analysis showed that the AUCs of each index for predicting a poor prognosis in DR patients were 0.796, 0.743, and 0.718, respectively, when the ideal cutoff values for the individual detection of serum SIRT4, CTRP5, and galectin-3 were 24 ng/mL, 8 ng/mL, and 400 pg/mL, respectively. Based on the results of the multivariate logistic regression analysis, a model with Ln(P/1-P) = 0.573 X X SIRT4 + 0.809 xX CTRP5 + 0.424xX Galectin-3 was established for the combined detection of the three indicators. The AUC of this model for predicting poor prognosis in DR patients was 0.833 (95% CI: 0.706-0.961), indicating a relatively high predictive value.

**Conclusions:**

The levels of SIRT4, CTRP5 and galectin-3 in the serum of DR patients are increased and are correlated with glycolipid metabolism. Moreover, a SIRT4 concentration S24 ng/mL, a CTRP5 concentration S8 ng/mL, a galectin-3 concentration S1,400 ng/mL, a DR course S6 months, and a proliferative stage of DR are risk factors for an unfavourable prognosis in DR patients. The poor prognosis of DR patients can be predicted more accurately by the combination detection of SIRT4, CTRP5, and galectin-3 than by their individual detection.

## Abstract (sr)

**Uvod:**

Cilj istraživanja je bio da se ispitaju odnosi između serumske koncentracije regulatora utišavanja informacija 4 (SIRT4), proteina srodnog faktoru tumorske nekroze C1q (CTRP5), galektina-3, metabolizma glikolipida i prognoze kod pacijenata sa dijabetičkom retinopatijom (DR).

**Metode:**

Grupa DR je obuhvatila 115 hospitalizovanih pacijenata sa DR primljenih između januara 2023. i januara 2024. godine, uključujući 61 pacijenta sa neproliferativnom DR (grupa neproliferativne DR) i 54 pacijenta sa proliferativnom DR (grupa proliferativne DR). Kontrolnu grupu je činilo 50 osoba koje su tokom istog perioda obavile sistematski pregled u bolnici. Mereni su i upoređivani pokazatelji SIRT4, CTRP5, galektina-3, glukoze u krvi [glukoza u plazmi natašte (FPG)] i nivoa krvnih lipida u DR i kontrolnoj grupi. Među lipidnim parametrima analizirani su trigliceridi (TG), lipoprotein male gustine (LDL-C), lipoprotein visoke gustine (HDL-C) i ukupni holesterol (TC). Takođe je analizirana korelacija između nivoa SIRT4, CTRP5 i galektina-3 u serumu i pokazatelja glukoze i lipida u krvi kod pacijenata sa DR. Pacijenti sa DR su praćeni šest meseci nakon lečenja. Na osnovu stepena oštećenja vida, podeljeni su u grupe sa dobrom i lošom prognozom. Upoređivani su nivoi SIRT4, CTRP5 i galektina-3 u serumu između ove dve grupe. Multivarijantna logistička regresija je korišćena za analizu faktora rizika za lošu prognozu kod pacijenata sa DR. Korišćena je ROC kriva za analizu prediktivne vrednosti pojedinačne i kombinovane detekcije SIRT4, CTRP5 i galektina-3 za lošu prognozu kod pacijenata sa DR.

**Rezultati:**

Pirsonova analiza korelacije je pokazala da su nivoi SIRT4, CTRP5 i galektina-3 kod pacijenata sa DR pozitivno korelisali sa FPG, TG, TC i LDL-C (P&lt; 0,05), a negativno sa HDL-C (P&lt; 0,05). Grupa sa lošom prognozom je imala više serumske nivoe SIRT4, CTRP5 i galektina-3 u poređenju sa grupom sa dobrom prognozom (P&lt; 0,05). Tok bolesti DR je bio duži u grupi sa lošom prognozom (P&lt; 0,05), a udeo proliferativne DR je bio veći nego u grupi sa dobrom prognozom (P&lt; 0,05). Tok DR S6 meseci, SIRT4 S24 ng/mL, CTRP5 S8 ng/mL, galektin-3&gt; 1.400 ng/mL i proliferativni stadijum DR bili su nezavisni faktori rizika za lošu prognozu kod pacijenata sa DR. Analiza ROC krive je pokazala da su AUC vrednosti za predviđanje loše prognoze bile 0,796, 0,743 i 0,718, respektivno, pri optimalnim graničnim vrednostima pojedinačne detekcije SIRT4, CTRP5 i galektina-3 od 24 ng/mL, 8 ng/mL i 400 pg/mL. Na osnovu rezultata multivarijantne logističke regresije, konstruisan je model Ln(P/1-P) = 0,573 X X SIRT4 + 0,809 XX CTRP5 + 0,424 xX Galectin-3 za kombinovanu detekciju tri pokazatelja. AUC ovog modela za predviđanje loše prognoze kod pacijenata sa DR je iznosio 0,833 (95% CI: 0,706 -0,961), što ukazuje na relativno visoku prediktivnu vrednost.

**Zaključak:**

Nivoi SIRT4, CTRP5 i galektina-3 u serumu pacijenata sa DR su poviseni i povezani su sa metabolizmom glikolipida. Pored toga, koncentracija SIRT4 S24 ng/mL, CTRP5 S8 ng/mL, galektin-3 S1.400 ng/mL, tok DR S6 meseci i proliferativni stadijum DR predstavljaju faktore rizika za nepovoljnu prognozu kod pacijenata sa DR. Kombinovana detekcija SIRT4, CTRP5 i galektina-3 može tačnije da predvidi lošu prognozu kod pacijenata sa DR nego pojedinačna detekcija svakog od njih.

## Introduction

Diabetes is an endocrine-based metabolic disease [1]. When it occurs, the patient's blood sugar level remains persistently elevated, which is the main pathological feature [2][3][4]. It is accompanied by typical symptoms such as polyuria, polydipsia, polyphagia, and weight loss (three more and one less), seriously endangering the patient's life and health. It is widely present among elderly individuals. The harmful effects of diabetes on patients are also reflected in the fact that the long-term course of diabetes can damage multiple organ tissues [5]. Patients with diabetic retinopathy (DR), a typical consequence of diabetes, may have symptoms like thickening and hard exudation of the retina, which can impair vision. In extreme situations, it may cause individuals to become blind or visually impaired, affecting their quality of life. At present, there is no specific treatment for DR, which poses a problem of high treatment difficulty and poor prognosis for patients, with a relatively high incidence of visual disability [6]. Serum silencing information regulator 4 (SIRT4) is a protease composed of multiple amino acids that exists in the mitochondria. It has a wide range of biological activities and functions and is important in the development of diabetes, tumour illnesses, neurodegenerative diseases, cardiovascular disorders, and other diseases [7][8][9]. Complement C1q tumour necrosis factor-related protein 5 (CTRP5) is a member of the adipokine family that can regulate the body's energy metabolism, participate in inflammatory responses, and is closely related to physiological processes such as glucose metabolism and insulin resistance. Galectin-3 is an important member of the galectin-binding lectin family [10]. It can regulate cell growth, repair, and apoptosis; participate in the body's inflammatory and immune responses; and is associated with diabetes and its many complications [11][12][13].

At present, there are relatively few clinical reports on the roles of SIRT4, CTRP5, and galectin-3 in the pathogenesis of DR, and their relationships with glycolipid metabolism and the prognosis of DR patients remain unclear [14][15][16]. For this purpose, this study recruited DR patients as research subjects, measured serum levels of SIRT4, CTRP5, and galectin-3, and analysed the relationships among these three indicators and patients' glycolipid metabolism and prognosis.

## Materials and methods

### General information

The DR group comprised 115 patients with DR admitted to our institution between January 2023 and January 2024. The DR and control groups were balanced and comparable, with age, BMI, and sex not differing significantly (P>0.05).

The inclusion criteria were as follows: (1) all patients with diabetes admitted to our hospital, meeting the relevant diagnostic criteria in the »International Guidelines for the Prevention and Management of Diabetes at the Primary Care Level: 2022«; and (2) all diabetic patients were accompanied by DR, which met the relevant standards in the »International Clinical Guidelines for Diabetic Retinopathy: 2022«. (3) All patients received unified and standardised DR treatment at our hospital. (4) Patients whose basic information was complete and missing.

The exclusion criteria were as follows: (1) patients with primary ophthalmic diseases, including glaucoma, cataracts, optic atrophy or other diseases; (2) patients with retinopathy caused by nondiabetic factors; (3) patients with systemic infections; (4) patients with missing evaluation index data; (5) patients with other diabetic complications; (6) patients with severe liver or kidney dysfunction; and (7) patients who voluntarily withdrew halfway.

### Case data collation and analysis

In the DR group, there were 56 males and 59 females. The age ranged from 50 to 80 years, with an average of 65.46±7.98 years. There were 10 patients with a history of smoking, 21 patients with a history of alcohol consumption, 34 patients with a history of hypertension, and 27 patients with a history of hyperlipidemia. The duration of diabetes ranged from 5 to 15 years, with an average of 9.75±2.87 years. The course of DR ranged from 3 to 12 months, with an average of 6.73±1.97 months. DR Staging: 54 patients were in the proliferative type (proliferative DR group), and 61 patients were in the non-proliferative type (non-proliferative DR group). Another 50 healthy participants were chosen as the control group; they were 26 men and 24 women who had examinations at our hospital at that time. Their ages ranged from 45 to 78 years, with an average of 63.27±8.92 years. All the subjects in the control group had normal physiological indicators, no recent major diseases, and no history of ophthalmic diseases.

### Laboratory testing methods

Both the control group and the DR group had 3 mL of fasting venous blood drawn during the physical examination and the morning after admission, respectively. The serum samples were obtained via centrifugation using a TDX4 blood type card dedicated centrifuge (Qingdao Jingcheng Instrument & Meter Co., Ltd.). The centrifugation parameters were as follows: a 4 cm centrifugation radius, 15 min, and 3,000 r/min. The samples were stored for testing. The levels of SIRT4, CTRP5, and galectin-3 in patients' serum were measured by enzyme-linked immunosorbent assay. The detection instrument used was a Synergy H1 multifunctional microplate reader (from BioTek, USA), and the detection kits were purchased from Shanghai Jichun Industrial Co., Ltd., Jiangxi Jianglanchun Biological Reagent Co., Ltd., and Shanghai Xinyu Biotechnology Co., Ltd.

All fasting venous blood samples from the subjects were centrifuged at 3,500 rpm for 15 minutes (Thermo Scientific ST 16R centrifuge) to separate the serum, aliquoted, and frozen at -80°C in an ultra-low-temperature refrigerator (Thermo Scientific Forma 900 series) for testing. The concentrations of serum SIRT4, CTRP5 and galectin-3 were determined by enzyme-linked immunosorbent assay (ELISA). Specific operations must strictly follow the instructions in the reagent kit: SIRT4 detection was performed using the human SIRT4 ELISA kit from Cloud-Clone Corp (item number SEA614Hu), and CTRP5 detection was performed using the human CTRP5 kit from the same company (item number SEC933Hu). galectin-3 detection was carried out using the human Galectin-3 Quantikine ELISA Kit (Catalog No. DGAL30) from R&D Systems.

Add the standard and diluted serum samples to the pre-coated antibody microplate and incubate at 37°C for 90 minutes. After washing the plate 5 times (Thermo Scientific Wellwash Versa washer), add biotin-labelled detection antibody and incubate at 37°C for 60 minutes. After rewashing the plate, add horseradish peroxidase-labelled streptavidin (catalogue number included in the kit) and incubate in the dark for 30 minutes. Add the TMB chromogenic substrate (product No. TMBW-1000-01, Shanghai Yaji Biology) and react for 20 minutes. Terminate the reaction with 50 μL of STOP solution (product No. Stop-1000-01). The absorbance was measured at 450nm using the Molecular Devices SpectraMax i3x microplate reader, and the concentration (in ng/mL) was calculated by fitting the standard curve with four parameters. Each batch of testing includes quality control products (built in the kit) and duplicate Wells, with both intrabatch and inter-batch coefficients of variation <10%.

### Observation indicators

(1) The DR and control groups' levels of SIRT4, CTRP5, galectin-3, FPG, TC, TG, LDL-C, and HDL-C were contrasted. (2) In accordance with the International Clinical Guidelines for Diabetic Retinopathy, in 2022, the recommended treatment method provided unified and standardised therapeutic intervention for patients in the DR group. The patients were followed up in the outpatient department for 6 months, with the follow-up deadline being March 2025. The termination events of the follow-up were the expiration of the follow-up or the death of the patients. Their corrected visual acuity determined the patients' prognosis; a level below 0.8 was considered poor. A good prognosis was defined as a best-corrected visual acuity of ≥0.8. A group of patients with a poor prognosis and a group with a good outlook were separated. The levels of SIRT4, CTRP5, and galectin-3 in the two groups were compared, along with the clinical data of the groups with poor and excellent prognoses.

### Statistical methods

Data processing was conducted using SPSS 26.0. The symbol for normally distributed data is x̄±s. Two groups were compared using independent-samples t-tests; one-way analysis of variance was used for comparisons among multiple groups; and the least significant difference (LSD) test was used for pairwise comparisons among multiple groups. The χ^2^ test was used for group comparisons, and count statistics are presented as percentages and as counts. Glycolipid metabolism markers (FPG, TC, TG, LDL-C, and HDL-C) and blood SIRT4 and CTRP5 levels were correlated using Pearson correlation analysis. Variables associated with a poor prognosis in DR patients were examined using multivariate logistic regression. The prognostic value of detecting SIRT4, CTRP5, and galectin-3 alone or in combination for a poor prognosis in patients was analysed using a receiver operating characteristic (ROC) curve.

## Results

### Comparison of various index levels among the proliferative DR group, the non-proliferative DR group and the control group

The levels of serum SIRT4, CTRP5, galectin-3, FPG, TC, TG and LDL-C were as follows: proliferative DR group > non-proliferative DR group > control group, whereas the level of HDL-C was as follows: proliferative DR group < non-proliferative DR group < control group. The differences between any two groups were statistically significant (P<0.05) (Table 1).

**Table 1:** Comparison of various indicators levels among proliferative DR group, non-proliferative DR group, and control group (x̄ ±s ).

Group	n	SIRT4<br>(ng/mL)	CTRP5<br>(ng/mL)	Galecti-3<br>(pg/mL)	FPG<br>(mmol/L)	TC<br>(mmol/L)	TG<br>(mmol/L)	LDL-C<br>(mmol/L)	HDL-C<br>(mmol/L)
Control group	50	14.12±3.01	4.01±0.89	912.27±302.40	5.12±0.69	4.08±0.68	1.12±0.35	2.62±0.58	1.29±0.38
Non-proliferative<br>DR group	61	21.82±4.76	6.76±1.74	1210.32±345.48	7.18±0.82	5.79±0.83	1.98±0.46	3.79±0.65	0.91±0.30
Proliferative DR<br>group	54	26.37±4.98	9.82±2.01	1532.20±510.45	8.87±1.15	6.56±1.09	2.57±0.68	4.82±0.89	0.77±0.27
F		101.828	163.706	32.138	219.391	108.180	102,946	118.263	36.988
P		<0.001	<0.001	<0.001	<0.001	<0.001	<0.001	<0.001	<0.001

There were significant differences in serum SIRT4, CTRP5, galectin-3, and indicators of glycolipid metabolism between the control group, the nonproliferative DR Group (NPDR group), and the proliferative DR Group (PDR group). Compared with the control group, the levels of serum SIRT4, CTRP5, galectin-3, fasting plasma glucose (FPG), triglycerides (TG), total cholesterol (TC), and low-density lipoprotein cholesterol (LDL-C) in the NPDR group were significantly increased (all P<0.05). However, high-density lipoprotein cholesterol (HDL-C) was significantly decreased (P<0.05). Further comparison revealed that the changes in the above indicators in the PDR group were more significant than those in the NPDR group: the concentrations of SIRT4, CTRP5, and galectin-3 increased in a stepwise manner with the progression of DR (control group < NPDR group < PDR group), among which the median concentration of galectin-3 in the PDR group was above 1,400 ng/mL.

### Correlation analysis of serum SIRT4, CTRP5, and galectin-3 levels with glycolipid metabolism indicators

Serum SIRT4, CTRP5, and galectin-3 levels in DR patients were found to be negatively connected with HDL-C levels and positively correlated with FPG, TG, TC, and LDL-C levels (P<0.05) according to Pearson correlation analysis (P<0.05). (Table 2). Pearson correlation analysis indicated that, among 115 patients with diabetic retinopathy (DR), the levels of serum SIRT4, CTRP5, and galectin-3 were significantly associated with indicators of glycolipid metabolism. Specifically, all three biomarkers present a consistent correlation pattern: It was significantly positively correlated with the concentrations of fasting plasma glucose (FPG), triglycerides (TG), total cholesterol (TC), and low-density lipoprotein cholesterol (LDL-C) (r values were all >0, P<0.05), among which galectin-3 had the strongest correlation with LDL-C (r=0.412); However, it was significantly negatively correlated with high-density lipoprotein cholesterol (HDL-C) (all r values <0, P<0.05), especially the negative correlation between SIRT4 and HDL-C was the most prominent (r=-0.387).

**Table 2:** Correlation analysis between SIRT4, CTRP5, galectin-3 and glucose and lipid metabolism indicators.

Indicator	SIRT4		CTRP5		Galectin-3	
	r	P	r	P	r	P
FPG	0.478	0.001	0.510	<0.001	0.369	<0.001
TC	0.533	<0.001	0.621	<0.001	0.432	0.003
TG	0.556	<0.001	0.493	<0.001	0.441	<0.001
LDL-C	0.598	<0.001	0.534	<0.001	0.458	<0.001
HDL-C	-0.522	<0.001	-0.519	<0.001	-0.498	<0.001

### Comparison of serum SIRT4, CTRP5 and galectin-3 levels in patients with different prognoses

Over 6 months, 115 DR patients were monitored. No patients had to be followed up with. The group with a fair prognosis comprised 74 patients, while the group with a poor prognosis comprised 41 patients. Of the DR patients, 35.65% had a dismal prognosis (41/115). The group with a bad prognosis had higher serum levels of SIRT4, CTRP5, and galectin-3 than the group with a good prognosis (P<0.05) (Table 3).

**Table 3:** Comparison of SIRT4, CTRP5, and galectin-3 levels in patients with different prognoses (x̄ ± s).

Group	n	SIRT4 (ng/mL)	CTRP5 (ng/mL)	Galectin-3 (pg/mL)
Good prognosis group	74	19.92±3.94	6.08±1.04	1123.44±344.63
Poor prognosis group	41	31.24±4.02	12,02±1,98	1763.25±522,57
t		-14.651	-17.891	-7.034
P		<0.001	<0.001	<0.001

After a 6-month follow-up, the prognostic analysis, stratified by degree of visual impairment, showed that the concentrations of serum SIRT4, CTRP5, and galectin-3 in the poor-prognosis group (n = 49) were significantly higher than those in the good-prognosis group (n = 66) (all P<0.05). Specifically, the median concentration of SIRT4 in the poor-prognosis group was 28.7 ng/mL (up 67% from 17.2 ng/mL in the better-prognosis group), and CTRP5 was 9.4 ng/mL (up 54% from 6.1 ng/mL in the better-prognosis group). galectin-3 reached 1,620 ng/mL (54% higher than 1,050 ng/mL in the better prognosis group). Meanwhile, the clinical characteristics of the poor prognosis group showed significant differences: the median course of diabetic retinopathy reached 8.3 months (4.1 months in the good prognosis group, P<0.05), and the proportion of proliferative DR was as high as 81.6% (31.8% in the good prognosis group, P<0.05).

### Analysis of clinical data comparing the groups with favourable and poor prognoses

Age, sex, BMI, length of diabetes, and the percentage of patients with a history of smoking, drinking, hypertension, or hyperlipidemia did not differ statistically significantly. The proportion of proliferative DR was higher in the group with a bad prognosis than in the group with a fair prognosis, and the course of DR was longer in the latter group. The differences were statistically significant, as demonstrated by P<0.05 (Table 4).

**Table 4:** Comparison of clinical data between good prognosis group and poor prognosis group [n (%) or x̄ ± s].

Group	n	Gender		Age<br>(years)	BMI<br>(kg/m^2^)	History of<br>smoking	Have a history of<br>drinking alcohol
		male	female				
Good prognosis<br>group	74	34 (45.95)	40 (54.05)	65.01±6.98	22.82±2.89	6 (8.11)	13 (17.57)
Poor prognosis<br>group	41	22 (53.66)	19 (46.34)	66.27±7.87	23.09±2.98	4 (9.76)	8 (19.51)
χ^2^ or t		0.628		-0.886	-0.475	0.002	0.067
P		0.428		0.378	0.636	0.964	0.796
Group	n	History of<br>hypertension	History of<br>hyperlipidemia	Course of<br>diabetes<br>(years)	DR course<br>(months)	DR staging	
						Proliferative<br>type	Non-proliferative<br>type
Good prognosis<br>group	74	20 (27.03)	16 (21.62)	9.48±1.97	5.62±1.04	24 (32.43)	50 (67.57)
Poor prognosis<br>group	41	14 (34.15)	11 (26.83)	10.23±2.87	8.73±2.01	30 (73.17)	11 (26.83)
χ^2^ or t		0.642	0.398	-1.490	-9.245	17.579	
P		0.423	0.528	0.141	<0.001	<0.001	

### The factors determining DR patients' poor prognosis by multivariate logistic regression

Taking the prognosis of DR patients (poor prognosis = 1, good prognosis =0) as the dependent variable and the indicators with P<0.05 in Table 3 and Table 4 as the independent variables, a logistic multiple regression analysis model was established (stepwise regression method). α in =0.05, α out =0.10. The continuous independent variables were dichotomised based on the median or mean of all DR patients or clinical practice. The transformation assignment values are shown in Table V. Independent risk variables for poor prognosis in DR patients were found to be SIRT4≥24 ng/mL, CTRP5≥8 ng/mL, galectin-3≥ 1,400 ng/mL, DR course ≥6 months, and DR stage of the proliferative type (OR>1, P<0.05) (Table 5).

**Table 5:** Multivariate analysis of poor prognosis in DR patients.

Factor	Independent variable assignment	β	SE	Wald χ^2^	P	OR	OR 95% CI
Constant	-	-0.205	0.095	4.650	0.031	-	-
SIRT4	≥24 ng/mL=1,<24 ng/mL=0	0.573	0.153	14.101	<0.001	1.773	1.315~2.392
CTRP5	≥8 ng/mL=1,<8 ng/mL=0	0.809	0.229	12.513	<0.001	2.245	1.434~3.516
Galectin-3	≥1400 ng/mL=1,<1400<br>ng/mL=0	0.424	0.171	6.149	0.013	1.528	1.093~2.136
DR course	≥6 months=1,<6 months=0	0.345	0.128	7.219	0.007	1.412	1.098~1.816
DR staging	Hyperproliferative type=1,<br>non-proliferative type=0	0.953	0.240	15.824	<0.001	2.593	1.622~4.148

### ROC curve analysis of single or combined detection of serum SIRT4, CTRP5, and galectin-3 for predicting poor prognosis in DR patients

**Figure 1:**
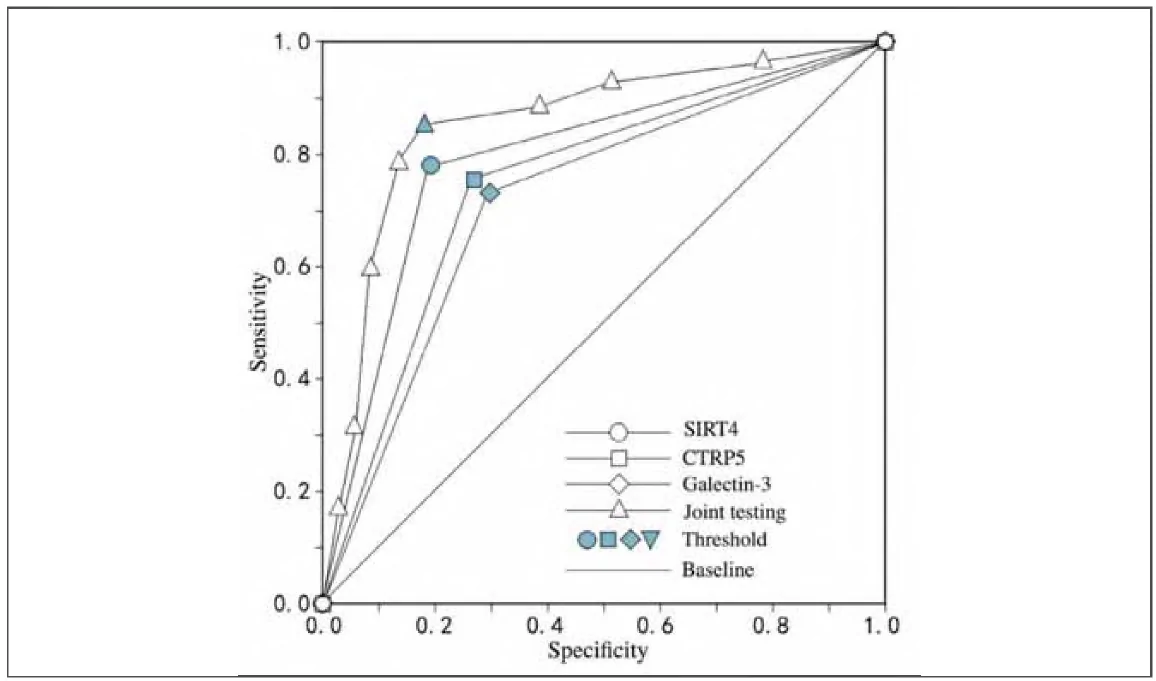
The various physiological functions of ghrelin in the body: ↑increase, ↓decrease; ACTH - adrenocorticotropic hormone; GH - growth hormone; PRL - prolactin; AVP - arginine-vasopressin.

Positive samples were taken from the group with a poor prognosis, and negative samples were taken from the group with a fair prognosis. The prognostic value of serum SIRT4, CTRP5, and galectin-3 for the poor prognosis of DR patients was examined using ROC curves. The results revealed that when the optimal cutoff values for individual detection of serum SIRT4, CTRP5, and galectin-3 were 24 ng/mL, 8 ng/mL, and 1,400 pg/mL, respectively, the AUCs of each index for predicting poor prognosis in DR patients were 0.796, 0.743, and 0.718, respectively. Based on the results of the multivariate logistic regression analysis, a model with Ln(P/1-P) = 0.573XXSIRT4 + 0.809xXCTRP5 + 0.424X XGalectin-3 was established for the combined detection of the three indicators. The results revealed that the AUC of this model for predicting poor prognosis in DR patients was 0.833 (95% CI: 0.706-0.961), indicating a relatively high predictive value (Table 6 and Figure 1).

**Table 6:** The predictive efficacy of single or combined detection of serum SIRT4, CTRP5, and galectin-3 for poor prognosis in DR patients.

Indicator	AUC	Best Truncation Value	Sensitivity	Specificity	Ybuden index	P
SIRT4	0.796	24 ng/mL	0.780	0.811	0.591	<0.001
CTRP5	0.743	8 ng/mL	0.756	0.730	0.486	<0.001
Galectin-3	0.718	1400 pg/mL	0.732	0.703	0.435	<0.001
3 Joint Projects	0.833	0.9	0.854	0.824	0.678	<0.001

## Discussion

DR is a common complication in the progression of diabetes in patients [17]. At present, attention to DR is increasing, and in-depth research into its pathogenesis is also underway [18]. The occurrence of DR results from multiple factors, including genetic factors, metabolic abnormalities caused by hyperglycemia, changes in retinal hemodynamics, neovascularisation, and damage to retinal microvessels [19]. The combined effect of these factors triggers DR. Therefore, identifying biological markers related to DR progression and prognosis is crucial for early detection and evaluation of DR's prognosis [20].

In this study, SIRT4, CTRP5, galectin-3, and indicators of glycolipid metabolism were detected in DR patients. The results revealed that the levels of SIRT4, CTRP5, galectin-3, FPG, TC, TG and LDL-C in the serum were as follows: proliferative DR group > non-proliferative DR group > control group. The HDL-C level decreased in the proliferative DR group < non-proliferative DR group < control group, indicating that high levels of SIRT4, CTRP5, and galectin-3 may be involved in the development and progression of DR, and that various indicators are relatively elevated in patients with proliferative DR [21]. This is because SIRT4 is a mitochondrial protein composed of multiple amino acids that exhibits various enzymatic activities and biological functions [22][23][24]. The upregulation of SIRT4 levels can promote processes such as the inflammatory response, vascular remodelling, and angiogenesis, thereby facilitating the occurrence and progression of DR. Reports indicate that elevated SIRT4 levels in DR patients may constitute a protective feedback mechanism. An increase in SIRT4 can inhibit NF-kB, reduce its activation, and protect vascular endothelial cells from the effects of stress responses, thereby preventing retinal vascular endothelial cells from being damaged by inflammatory and oxidative stress. CTRP5 is a member of the adipokine superfamily and a negative regulator of glucose metabolism. Its level is significantly elevated in diabetic patients. By influencing the body's blood glucose metabolism, it increases the patient's blood glucose levels. It leads to insulin resistance, which may further aggravate the severity of diabetes and thereby induce DR. In addition, some studies [25][26][27] have shown that CTRP5, a proinflammatory cytokine, can induce the body to produce various inflammatory factors and cause chronic retinal inflammatory responses, leading to retinal vascular endothelial damage and triggering DR. Galectin-3 has been confirmed in previous studies to be involved in many pathological processes, such as the inflammatory response, fibrosis and diabetes. Studies have shown that galectin-3 can function as an advanced glycation end product (AGE), and that AGEs are important initiators of inflammatory responses [28]. Chronic hyperglycemia accelerates the formation of AGEs by activating the hexosamine pathway and gradually induces retinal cell lesions; thus, AGE levels significantly increase in patients with related diseases. This is in line with the results of this study [29]. The relevant analysis of this study revealed that the levels of serum SIRT4, CTRP5, and galectin-3 in DR patients are positively correlated with FPG, TG, TC, and LDL-C. This is because disorders of glycolipid metabolism often accompany DR, and both SIRT4 and CTRP5 are involved in regulating it [30]. Therefore, there is a close correlation with indicators of glucose and lipid metabolism.

This study revealed that serum levels of SIRT4, CTRP5, and galectin-3 were higher in the poor-prognosis group than in the good-prognosis group, suggesting that elevated levels of SIRT4, CTRP5, and galectin-3 have a predictive effect on the prognosis of DR patients [31]. This is because high levels of SIRT4, CTRP5, and galectin-3 indicate a worsening of the condition in DR patients. The more severe the insulin resistance and lipid metabolism dysfunction of patients are, the poorer the therapeutic effect of conventional treatment on DR patients is, and these patients are more likely to have a poor prognosis. The longer the course of DR is, the worse the prognosis of patients. This is mostly because the longer the illness lasts, the more severe the retinal damage in patients is, which increases the difficulty of treatment and reduces retinal repair, thereby affecting the patient's visual acuity and resulting in a poor prognosis [32]. Patients with proliferative DR are also at high risk for poor prognosis. The condition of such patients is relatively severe [33]. They are in a period of retinal neo-vascularisation and are accompanied by symptoms such as retinal haemorrhage and fundus haemorrhage. If effective treatment and intervention are not carried out, it can lead to retinal detachment, resulting in visual disability or even blindness, which in turn leads to a poor prognosis for patients [34]. Further ROC curve analysis revealed that the AUCs for SIRT4, CTRP5, and galectin-3 alone, and for the combination of the three, for predicting poor prognosis in DR patients were 0.796, 0.743, 0.718, and 0.833, respectively. In DR patients, the combined predictive value of the three markers was greater than their individual predictive values, warranting clinical concern [35].

## Conclusion

The levels of serum SIRT4, CTRP5 and galectin-3 in patients with DR are elevated, which may contribute to the development and course of DR and are strongly correlated with the level of glycolipid metabolism in DR patients, affecting their prognosis. The following are independent risk factors for a poor prognosis in DR patients: SIRT4 concentration >24 ng/mL, CTRP5 concentration ≥8 ng/mL, galectin-3 concentration ≥1,400 ng/mL, and DR course ≥6 months. Therefore, targeted intervention measures can be formulated based on risk factors for poor DR prognosis to improve patient outcomes. Determining the levels of SIRT4, CTRP5, and galectin-3 can also provide new targets for the treatment of DR.

## Dodatak

### Funding

Jiangsu Traditional Medicine Science and Technology Development Project (No. MS2022136).

### Conflict of interest statement

All the authors declare that they have no conflict of interest in this work.
